# Potash fertilizer promotes incipient salinization in groundwater irrigated semi-arid agriculture

**DOI:** 10.1038/s41598-020-60365-z

**Published:** 2020-02-28

**Authors:** Sriramulu Buvaneshwari, Jean Riotte, Muddu Sekhar, Amit Kumar Sharma, Rachel Helliwell, M. S. Mohan Kumar, J. J. Braun, Laurent Ruiz

**Affiliations:** 1 Indian Institute of Science, Bangalore, India; 2 Indo-French Cell for Water Sciences, Indian Institute of Science, Bangalore, India; 3 IRD, CNRS, UPS, UMR GET, Toulouse, France; 4 INRAE, AGROCAMPUS OUEST, UMR SAS, Rennes, France; 5 Univ Rennes, CNRS, UMR LETG, Rennes, France; 6 James Hutton Institute, Aberdeen, United Kingdom; 7 Present Address: ICWaR, Indian Institute of Science, Bangalore, India

**Keywords:** Hydrology, Environmental chemistry, Environmental impact

## Abstract

Incipient groundwater salinization has been identified in many arid and semi-arid regions where groundwater is increasingly used for irrigation, but the dominant processes at stake in such context are yet uncertain. Groundwater solutes originates from various sources such as atmospheric inputs, rock dissolution and fertilizer residues, and their concentration is controlled by hydrological processes, in particular evapotranspiration. Here, we propose a deconvolution method to identify the sources and processes governing the groundwater Chloride concentration in agricultural catchments, using the relative variations of Sodium and Chloride and using a neighbouring pristine catchment as a reference for the release rate of Na by weathering. We applied the deconvolution method to the case of the Kabini Critical Zone Observatory, South India, where groundwater was sampled in 188 farm tubewells in the semi-arid catchment of Berambadi and in 5 piezometers in the pristine catchment of Mule Hole. In Berambadi, groundwater composition displayed a large spatial variability with Cl contents spanning 3 orders of magnitude. The results showed that the concentration factor due to evapotranspiration was on average about 3 times more than in the natural system, with higher values in the valley bottoms with deep Vertisols. Linked with this process, large concentration of Chloride originating from rain was found only in these areas. At the catchment scale, about 60 percent of the Chloride found in groundwater originates from fertilizer inputs. These results show that Potassium fertilization as KCl is an important source of groundwater salinization in semi-arid context, and stress that identifying dominant drivers is crucial for designing efficient mitigation policies.

## Introduction

Groundwater salinization is a growing global concern^1^. The general processes leading to salinization are well understood since a long time^2^ with causes being either natural, anthropogenic or a mix of the two^3^. Indeed, most of the literature has been focused on areas prone to widespread salinization, such as coastal areas prone to seawater intrusion^4–6^, groundwater irrigation from aquifers bearing evaporites^7^, command areas of dams where waterlogging restrict salt leaching^8^ or arid areas where historical accumulation of salts in the deep vadose zone due to evapotranspiration can be leached when natural vegetation is replaced by agriculture^9^. However, even in the absence of such adverse conditions, risks of groundwater recharge salinization have been identified in many arid and semi-arid regions where groundwater is increasingly used for irrigation^10–13^. As in these situations the attention is directed towards water resource depletion issues, this incipient salinization has been scantly studied^14^. As of now, the extent of the problem and its spatial variability is hardly documented and designing sustainable management practice to mitigate it is challenging because the relative contributions of the potential processes at stake are difficult to assess.

Salts in the soil naturally originate from precipitation and mineral dissolution. Their concentrations are primarily linked to the ratio between evapotranspiration (ET) and precipitation (P), explaining why high salt concentrations in soils are mainly found in arid and semi-arid regions^9^. Chlorine is one of the most abundant elements in rain water, but is rarely present in primary minerals, except from evaporites. This feature, combined with its conservative behaviour, makes it a useful tracer of water balance, and it has been widely used to estimate groundwater recharge with Chloride Mass Balance (CMB) method^15–21^. Like Chlorine, Sodium is abundant in rain water, but it is also released by Na-plagioclase weathering^22,23^. The Na/Cl ratio is therefore widely used to characterize water types, with surface waters being close to marine ratio (0.86) while water having much higher ratios indicate long residence time in weathering material^7,24–26^.

In agrosystems, salts are also often added by fertilizers. In many agrosystems Cl is widely applied as potash, i.e. in the form of KCl^27^. Amongst the mineral fertilizers, KCl has the highest impact on soil salinity because K is efficiently taken up and exported by plants as a macronutrient while Cl, which is a micro-nutrient^28^ remains in soil pore water and concentrates through evapotranspiration. Chloride toxicity is commonly observed in arid and semiarid regions where improper irrigation practices^29^ and high evaporation rates can lead salt accumulation in soils^30^. To the contrary, Na is rarely present in chemical fertilizers since nitrogen fertilization with NaNO_3_ was abandoned in the early twentieth century^31^. Therefore, in intensive agrosystems, fertilizer addition might affect the interpretation of Na/Cl ratios and the estimation of recharge with CMB.

Agriculture is also impacting the salt concentration in soil, by modifying evapotranspiration (ET) rates. Rainfed agriculture, with long periods of bare soils, has usually a lower ET than natural vegetation, leading to diluted soil water concentrations^9^. To the contrary, irrigation increases ET by allowing longer crop cycles and denser canopies, leading to increased salt concentration in soil pore water. In the case of GW irrigation, recycling of salts is likely to worsen this problem^32–34^.

In India, increasing groundwater irrigated area has allowed to sustain the growth of agricultural production since 40 years and to meet the need of a growing population^11,35^. In the Deccan plateau in South India, the development of submersible pump technology allowed millions of small farmers, typically holding about 1 ha of land, to access irrigation through tubewells in a region previously dominated by rainfed agriculture^36^. The resulting high diversity of cropping systems and irrigation techniques is likely to induce a large variability in groundwater recharge composition. For instance, the variability of groundwater contamination by nitrates was revealed at the scale of a small agricultural watershed from high density tubewell monitoring and attributed to the heterogeneity of aquifer properties and farming practices^37^. In the same site^38^, showed that incipient groundwater salinization was already significantly affecting crop yields, but the processes driving the spatial variations of salinization in groundwater have still to be documented.

Existing approaches to identify hydro chemical processes in groundwater are poorly adapted to such a context, characterized by high small-scale spatial heterogeneity. Geochemical analysis^39^ (e.g. piper diagram or multivariate analysis) are well suited for large aquifers, where large scale spatial heterogeneity in geological substrate or land use create strongly differentiated geochemical signatures in groundwater. Similarly, geochemical tracers, like isotopes, are widely used^40,41^. Although being more quantitative, they also need a strong spatial signal (e.g. altitudinal gradient in the case of water isotopes). Using mechanistic modelling would be suitable, and a wide range of models exist^42^ but it would require gathering large datasets to parametrize such heterogeneous systems. Deconvolution methods are classically used to identify the origin of solutes in rivers^25,43–47^and Na/Cl ratio is commonly used to assess the respective contributions of atmospheric vs geogenic sources of solutes and estimate silicate weathering and particularly Na-plagioclase weathering^48^. However, the contribution of fertilizer inputs is usually neglected in such studies.

As the consequence, identifying and quantifying the sources and processes driving salinization in groundwater irrigated agrosystems remains a challenge. In this paper, we propose to combine data from an agricultural catchment (Berambadi) and a neighbouring pristine forested catchment (Mule Hole), both belonging to the Kabini Critical Zone Observatory in South India^49^ to (i) explore the spatial variability of groundwater chemical composition based on high density tubewell sampling and (ii) quantify the concentration factor (CF) due to Evapotranspiration (ET) and estimate the relative contribution of rainfall vs fertilizer addition to Cl total input in agrosystems.

## Rationale of the Deconvolution Method

In agrosystems, solutes originate from atmospheric inputs, mineral weathering and fertilizer residues, and their concentration in groundwater increase with the effect of evapotranspiration. In our method, we propose to deconvolute groundwater Chloride concentrations using Na as a reference to assess the contribution of fertilizers to incipient salinization in agricultural catchments.

In a natural system, the only source of Cl is rain^47,50,51^ and therefore we can write:

1 $${[{\rm{Cl}}]}_{{\rm{G}}}={[{\rm{Cl}}]}_{{\rm{G}}({\rm{R}})}$$

where [Cl]_G_ = Chloride concentration in groundwater (µmolL^−1^), [Cl]_G(R)_ = concentration in groundwater of Chloride originating from rain input (µmolL^−1^).

As Cl is conservative in the system and plant uptake is negligible, the ratio between the Cl concentration in groundwater and in the rain can be used to estimate the concentration factor due to ET^52^ (CF, dimensionless). For a given tubewell, we can write:

2 $${\rm{CF}}=\frac{{[{\rm{Cl}}]}_{{\rm{G}}}}{{[{\rm{Cl}}]}_{{\rm{R}}}}$$

where [Cl]_R_ = Chloride concentration in rain (µmolL^−1^)

For Na, as it is added both by the rain and by mineral weathering, we can write

3 $${[{\rm{Na}}]}_{{\rm{G}}}={[{\rm{Na}}]}_{{\rm{G}}({\rm{R}})}+{[{\rm{Na}}]}_{{\rm{G}}({\rm{w}})}$$

where [Na]_G_ = Sodium concentration in groundwater (µmolL^−1^), [Na]_G(R)_ = Concentration in groundwater of Sodium originating from rain input (µmolL^−1^), ${[{\rm{Na}}]}_{{\rm{G}}({\rm{w}})}$ = Concentration in groundwater of Sodium originating from weathering (µmolL^−1^).

If we assume that dissolved Na is conservative in the system, we can write:

4 $$\frac{{[{\rm{Na}}]}_{{\rm{R}}}}{{[{\rm{Cl}}]}_{{\rm{R}}}}=\frac{{[{\rm{Na}}]}_{{\rm{G}}({\rm{R}})}}{{[{\rm{Cl}}]}_{{\rm{G}}}}=\frac{{[{\rm{Na}}]}_{{\rm{G}}}-{[{\rm{Na}}]}_{{\rm{G}}({\rm{w}})}}{{[{\rm{Cl}}]}_{{\rm{G}}}}$$

where [Na]_R_ = Sodium concentration in rain (µmolL^−1^)

The concentration in groundwater of Sodium originating from weathering ${[{\rm{Na}}]}_{G(w)}$ can hence be estimated from Eqs. 3 and 4 as:

5 $${[{\rm{Na}}]}_{{\rm{G}}({\rm{w}})}={[{\rm{Na}}]}_{{\rm{G}}}-{[{\rm{Na}}]}_{{\rm{R}}}\frac{{[{\rm{Cl}}]}_{{\rm{G}}}}{{[{\rm{Cl}}]}_{{\rm{R}}}}={[{\rm{Na}}]}_{{\rm{G}}}-{[{\rm{Na}}]}_{{\rm{R}}}\,{\rm{CF}}$$

Finally, if we consider that the production of Na by plagioclase weathering takes place mostly in the root zone, i.e. soil and shallow saprolite^51^, the concentration factor due to ET will be the same as for Na inputs from rain. Therefore, we can estimate the input concentration of Sodium originating from weathering ${[{\rm{Na}}]}_{{\rm{w}}}$ (µmolL^−1^) as follow:

6 $${[{\rm{Na}}]}_{{\rm{w}}}=\frac{{[{\rm{Na}}]}_{{\rm{G}}({\rm{w}})}}{{\rm{CF}}}$$

In agrosystems, Cl is not only added by rain but also often by fertilizers, mostly in the form of KCl. Cl and Na concentrations in groundwater can be decomposed as:

7 $${[{\rm{Cl}}]}_{{\rm{G}}}={[{\rm{Cl}}]}_{{\rm{G}}({\rm{R}})}+{[{\rm{Cl}}]}_{{\rm{G}}({\rm{F}})}$$

8 $${[{\rm{Na}}]}_{{\rm{G}}}={[{\rm{Na}}]}_{{\rm{G}}({\rm{R}})}+{[{\rm{Na}}]}_{{\rm{G}}({\rm{w}})}$$

where [Cl]_G(F)_ = Chloride concentration in groundwater originating from fertilizer input (µmolL^−1^)

If we assume that ${[{\rm{Na}}]}_{w}$ is similar in the natural system and the agrosystem and that the concentrations of Na originating from rain and weathering are affected similarly by evapotranspiration, we can use the average value of ${[{\rm{Na}}]}_{w}$ found in the natural system to estimate CF for each tubewell in the agrosystems as:

9 $${\rm{CF}}=\frac{{[{\rm{Na}}]}_{{\rm{G}}}}{{[{\rm{Na}}]}_{{\rm{R}}}+{[{\rm{Na}}]}_{{\rm{w}}}}$$

Assuming that both dissolved Cl and Na are conservative, we can write:

10 $$\frac{{[{\rm{Na}}]}_{R}}{{[{\rm{Cl}}]}_{{\rm{R}}}}=\frac{{[{\rm{Na}}]}_{G(R)}}{{[{\rm{Cl}}]}_{{\rm{G}}({\rm{R}})}}$$

which is equivalent to

11 $${[{\rm{Cl}}]}_{{\rm{G}}({\rm{R}})}={[{\rm{Na}}]}_{{\rm{G}}({\rm{R}})}\frac{{[{\rm{Cl}}]}_{{\rm{R}}}}{{[{\rm{Na}}]}_{{\rm{R}}}}$$

Using the above equations, we can calculate the contribution of rain [Cl]_G(R)_ and fertilizers [Cl]_G(F)_ to the Cl concentration in GW as:

12 $${[{\rm{Cl}}]}_{{\rm{G}}({\rm{R}})}=({[{\rm{Na}}]}_{G}-{[{\rm{Na}}]}_{{\rm{G}}({\rm{w}})})\frac{{[{\rm{Cl}}]}_{R}}{{[{\rm{Na}}]}_{{\rm{R}}}}=({[{\rm{Na}}]}_{G}-{[{\rm{Na}}]}_{w}{\rm{CF}})\frac{{[{\rm{Cl}}]}_{R}}{{[{\rm{Na}}]}_{{\rm{R}}}}$$

13 $${[{\rm{Cl}}]}_{{\rm{G}}({\rm{F}})}={[{\rm{Cl}}]}_{{\rm{G}}}-{[{\rm{Cl}}]}_{{\rm{G}}({\rm{R}})}$$

Finally, we can also calculate ${[{\rm{Cl}}]}_{F}$ (µmolL^−1^), being the input concentration of Chloride originating from fertilizers, i.e. without the concentrating effect of evapotranspiration, as follow:

14 $${[{\rm{Cl}}]}_{{\rm{F}}}=\frac{{[{\rm{Cl}}]}_{{\rm{G}}({\rm{F}})}}{{\rm{CF}}}$$

For the deconvolution method to be applicable, three main assumptions have to be verified, 1) dissolved Na and Cl are conservative 2) their concentrations are affected similarly by evapotranspiration and 3) mineral weathering rates in agricultural catchments can be deduced from neighbouring pristine catchments. In this study, we assessed these assumptions in the case of an agricultural catchment in South India (Berambadi) by:

1. Using previous studies in the site suggesting that Chloride and Sodium are conservative in this system and likely to be similarly affected by evapotranspiration.
2. Testing the assumption that the release of Na in groundwater by Na- plagioclase weathering occurs with the same intensity in both catchments (i.e. ${[{\rm{Na}}]}_{w}$ is similar). Indeed, variations in plagioclase weathering across wells and sites might occur due to differences in local composition of the bedrock, regolith thickness, residence time of the water in the vadose zone, and the aggressiveness of the groundwater^53^. To assess the variability in weathering rates between the two catchments, we used Europium, a Rare Earth Element which is more concentrated in plagioclase compared to the other REE. Its relative enrichment in groundwater with respect to its neighbouring REEs, Sm and Gd, induces a positive Europium anomaly (further notes as Eu*) and can be used as a proxy of relative plagioclase weathering in the vadose zone^54,55^. We compared the positive Eu-anomaly in the groundwater of both pristine and cultivated systems, assuming that a similar average and variability of the positive Eu-anomaly in groundwater for both sites would indicate a similar Na production by plagioclase weathering.

Finally, to assess the robustness of the method, we assess its sensitivity to the [Na]_w_ value used for the deconvolution in the agrosystems, using a range corresponding to the variability observed in the pristine catchment.

## Study Area

**Figure 1:**
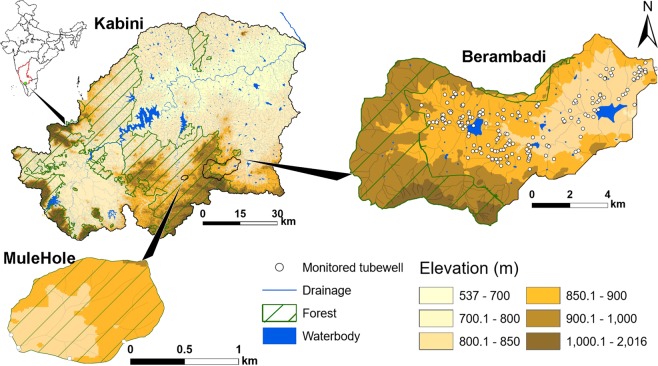
Elevation map of Kabini basin, which includes the Mulehole forested pristine catchment and Berambadi cultivated catchment, showing the density of monitored tube wells (maps were generated using ArcGIS version 10.3.0, https://desktop.arcgis.com/en/).

The deconvolution method was applied in the Kabini Critical Zone Observatory^56^ (M-TROPICS, part of OZCAR research infrastructure) in Southern India. The Observatory comprises the agricultural catchment of Berambadi and the neighboring pristine catchment of Mule Hole (Fig. 1). The main characteristics of the observatory are described in^49^.

The climate is tropical sub-humid. The monsoon dynamics drives three main seasons: Summer (dry season, from January to May), Kharif (South-West monsoon season, June to September) and Rabi (North-East monsoon season, from October to December). Rainfall patterns display broad decennial trends as well as strong inter-annual variability^57^ with recurrence of droughts^58^. The bedrock of both catchments consists in Precambrian gneiss with few patches of amphibolite, with a two layers aquifer: a regolith, composed of an immature saprolite (on average 15 m thick^23^; topped by a 2 m thick ferralsol/vertisol system^59^ and below a fissured zone located in the fresh bedrock. This structure is typical of a hard rock aquifer^60,61^. Vertisols are predominantly found in valley bottoms. Both saprolite and soils contain residual primary minerals like quartz, Na-plagioclase and sericite and secondary minerals like Fe-oxyhydroxides and clay minerals.

### The pristine, forested catchment of Mule Hole

The Mule Hole catchment (4.1km^2^) is located 11 °430N–76 °260E, about 10 km at the west of the Berambadi catchment, in the sub-humid zone of the climatic gradient induced by the western Ghats. This forested catchment is part of the Bandipur National Park and then preserved from any anthropogenic activity since at least the creation of the sanctuary in 1974. Long term annual rainfall ranges from 800 to 1500 mm/yr with an average of 1100 mm/yr. Vegetation consists of a dry deciduous forest dominated by the “ATT” facies, i.e. *A. latifolia*, – *T. grandis* and *T. alata* with *T. triendra* grass (Elephant grass)^51^. This experimental catchment is monitored for meteorological, hydrological and geochemical variables since 2003 for assessing, from decennial hydrological and geochemical budgets, the processes governing soil-plant-water interactions. Water mass balance indicates that evapotranspiration accounts for 80 to 90% of the water budget. As a consequence, the forest mediates through the water stock in the vadose zone, the groundwater recharge and discharge and the stream fluxes^19,62^. By combining the SVAT model COMFORT^62^ with the observations of tree growth, we recently established that co-dominant tree species (among which ATT species) display distinct root uptake depths, indicating that competition drives hydrological niche separation^63^. Deep root uptake explains the long residence time of water in the vadose zone of the forest, up to 20 years according to the COMFORT model^47^, depending on saprolite thickness and water content.

### The cultivated catchment of Berambadi

In the Berambadi catchment (84 km²) the climate is slightly drier than in Mule Hole. The average rainfall is 800 mm/year with a slight gradient from West to East from 900 to 750 mm/year. Most of the rainfall occurs during the southwest monsoon from June to September. Potential evapotranspiration is 1100 mm (aridity index P/PET of 0.7). Agricultural cropland and forest cover are the major land use in the catchment with 52% and 32%, respectively^64^. The development of tube well irrigation since 30 years has induced a shift from the rainfed to irrigated agriculture. As a result, the area with high water demanding cash crops (Turmeric, Banana, Sugarcane) increased at the expense of traditional rainfed crops such as finger millet, pulses etc. As groundwater availability for irrigation is limited by the low transmissivity of the aquifer, and by the fact that electricity for submersible pumps, although freely provided by the government, is only available for 3–4 h/day, the tube well density is increasing in the catchment^37^. About 5000 farms exist in the catchment, with an average size of about 1 ha, divided into cultivated plots with an average size of 0.2 ha^64,65^. This lead to a large diversity of agricultural practices, depending on crop and farm types. The diversity of farming systems across the catchment was characterized as three main farm types: High productive farms, small and marginal rainfed farms and small irrigated farms^65^.

## Materials and Methods

### Catchment monitoring and sampling

In the Mule Hole sub-catchment, we used 5 piezometers, drilled in 2003 and 2004 and located along the catchment boundaries, while in the Berambadi catchment we used 188 farmer’s tube wells (Fig. 1). Depths to groundwater (distance from ground surface to groundwater table static level, expressed in meter below the surface) was measured with a manual piezometric level sensor (skinny dipper device, Haron instruments). In the Berambadi catchment, measurements were done at least 10 hours after pumping stops, to allow water table to recover from drawdown and approach static level. In Mule Hole we collected the 5 piezometers on monthly basis from 2005 to 2018 while in Berambadi we sampled once the 188 tube wells, between 25^th^ to 28^th^ April 2014.

### Chemical analyses

Conductivity and pH were determined in the field using a WTW meter. Each sample was filtrated in the field and stored in two pre-cleaned polypropylene bottles. The first bottle, not acidified, was used for determination of major dissolved species. Anions and cations were measured with an Ion Chromatograph Metrohm 861. Silica concentration was determined using the molybdate blue method with a UV- visible Knauer detector. The accuracy of analyses was controlled with multiple Certified Reference Materials (AnionWS, ION-96.4, ION 915, SUPER-05, BIG MOOSE 02 and PERADE) depending on the range of concentrations. Alkalinity was determined with the alkalinity titrator Mettler Toledo-DL50 Rondolino. Usual precision obtained on major dissolved species determination was about 5%. Whole accuracy was also checked using NICB (Normalized Inorganic Charge Balance^66^. The other bottle was acidified onsite with bi-distilled nitric acid for determination of trace elements concentrations (including Al and Rare Earth Elements) with an Agilent 6000 quadripolar ICPMS at Geosciences Environment Toulouse (France). Accuracy of analyses was checked with SLRS 5 reference materials. Overall precision on trace element analyses was about 10%.

### Calculation of indices and mapping

The degree of pollution of groundwater was calculated according to the definition of^67^ which is based on the relative proportion of anions of possible anthropogenic origin compared to the whole anionic charge:

15 $$ \% \,{\rm{pollution}}=\frac{[{{\rm{Cl}}}^{-}]+[{{\rm{SO}}}_{4}^{2-}]+[{{\rm{NO}}}_{3}^{-}]}{[{{\rm{Cl}}}^{-}]+[{{\rm{SO}}}_{4}^{2-}]+[{{\rm{NO}}}_{3}^{-}]+[{\rm{Alkalinity}}]}\,\ast \,100$$

where all the concentrations are in μeq/L.

The Europium anomaly (Eu*) is a proxy for plagioclase weathering^54,55^. It is calculated by normalization of its concentration by those of his neighbours (Sm & Gd) and to the average gneissic bedrock^23^ according to the equation:

16 $$E{u}^{\ast }=\frac{\frac{E{u}_{GW}}{E{u}_{bedrock}}}{{(\frac{S{m}_{GW}}{S{m}_{bedrock}})}^{0.5}.{(\frac{G{d}_{GW}}{G{d}_{bedrock}})}^{0.5}}$$

The saturation index of groundwater regarding Na-plagioclase (albite) was calculated using the PHREEQC software^68^. Catchment water quality maps were constructed by interpolation of the borewell observation data using kriging and an exponential variogram model – characterizing the spatial dependence of the variability in MATLAB^69^ and displayed with ArcGIS version 10.3.0. It should be noted that uncertainty increases towards the boundaries of the mapped area.

Map of irrigation distribution was constructed from the results of ^64,70^. These authors used multi-temporal Landsat satellite images from the year 1990 to 2016. Details of the methods can be found in their paper. In brief, the irrigated area and non-irrigated classification were performed with Support vector machine algorithm using EVI, NDMI, and NDVI indices. The irrigated and non-irrigated croplands were estimated for the rabi and summer season at an interval of 5years with high classification accuracy (kappa coefficient greater than 0.9). No analysis was done during the monsoon (kharif) as is it mostly cloudy. In this paper, based on these data, we recomputed the map of irrigation distribution corresponding to the period anterior to the sampling date (1990–2013).

## Results

### Groundwater quality

In the pristine catchment of Mule Hole, conductivity ranges from 300 to 900 µS/cm and it is lithology controlled^23,71^. Highest conductivities are located in amphibolite bedrock, but the occurrence of this lithology is not frequent (7%) in this gneiss-dominated catchment. pH is neutral and rather homogeneous across the catchment (7.06 ± 0.2). Cation composition is dominated by Na and Ca. The cationic charges are mainly balanced by alkalinity, which accounts for up to 90% of anionic charges and to a lesser extent by chloride. Pollution Index was 11 ± 3% which is consistent with the pristine characteristics of Mule Hole.

**Figure 2:**
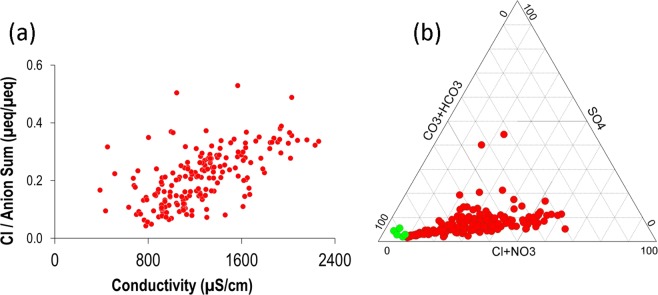
(**a**) Relationship between conductivity and relative proportion of chloride in Berambadi groundwater, (**b**) Evolution of anion composition from bicarbonate-dominated waters in the pristine end-member (Mule Hole, green dots) to Cl-and NO_3_-dominated waters in Berambadi (red dots).

In contrast, about 90% of Berambadi groundwater samples exhibit conductivities between 700 and 2000 µS/cm. Extreme values reach 2300 µS/cm, similar to those observed in the semi-arid part of the upper Cauvery Basin^72^. The anionic composition evolves as the conductivity increases, with an increase of Cl proportion (Fig. 2a) at the expense of alkalinity (Fig. 2b). Contrary to anions, none of the relative cation concentrations increases with conductivity (not shown). For a majority of samples, the cationic load is dominated by Na, followed by Ca and Mg. Potassium, although massively applied as fertilizer, never accounts for more the 2% of the cationic load. No relationships were found between conductivity and the concentration of any specific cations. Pollution index spans a large range of values, from 10% (similar to those of Mule Hole) to 75%, and displays a weak positive correlation with electrical conductivity (R^2^ = 0.34; not shown).

**Figure 3:**
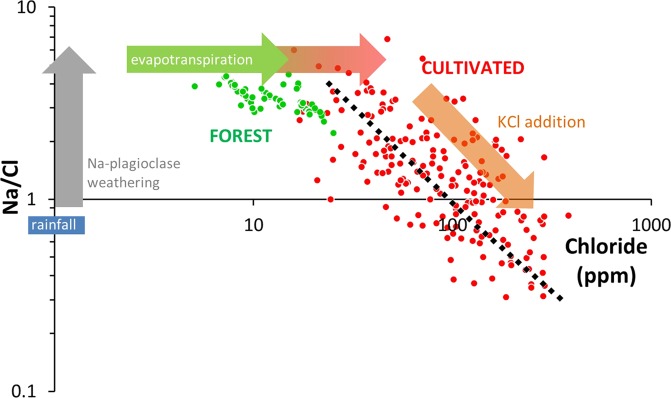
Relationship between Cl concentration and Na/Cl molar ratio in Berambadi (red dots) and Mule Hole (Green dots) groundwater. Dashed black line correspond to theoretical evolution of Na/Cl and Cl concentration if KCl is added to a pristine water. For clarity, only samples collected in 2014 in the Mule Hole catchment are shown.

The comparison between Na/Cl molar ratio and Cl concentration of groundwater samples (Fig. 3) reveals a contrasted pattern between pristine and agricultural catchments, which is in accordance with the assumptions on the processes controlling the relative evolution of Na and Cl in the groundwater posed in section 2.

In Mule Hole, local precipitations exhibit an average Na/Cl molar ratio close to the seawater value (0.85) and Cl concentration around 1 ppm^51^. Once infiltrated into the vadose zone, Cl concentration rises due to evapotranspiration only, while Na concentration also rises due to Na-plagioclase weathering. As a consequence, the Na/Cl molar ratio in groundwater increases to reach an average of 3.5 ± 0.98. In Berambadi, for groundwater sample with similar Na/Cl ratios as in Mule Hole, the Cl concentration is higher, suggesting a higher rate of evapotranspiration. In addition to the above processes, KCl addition leads to an increase of Cl concentration and a concomitant decrease of the Na/Cl ratio down to 0.3.

**Figure 4:**
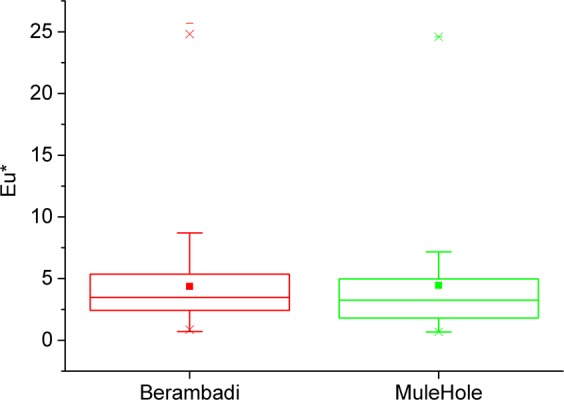
Europium anomaly variation in Berambadi and Mulehole groundwater.

The average and standard deviations of the Europium anomaly Eu* (Fig. 4) in groundwater are similar in Mule Hole (4.0 ± 4.6) and in Berambadi (4.4 ± 3.3) despite Na concentration being on average larger by a factor 4 in Berambadi compared to Mule Hole. This suggests that plagioclase weathering rates are similar in both catchments, and therefore the input concentration of Sodium originating from weathering ${[{\rm{Na}}]}_{w}$ estimated in Mule Hole can be used to apply the deconvolution method in Berambadi.

### Deconvolution method

**Figure 5:**
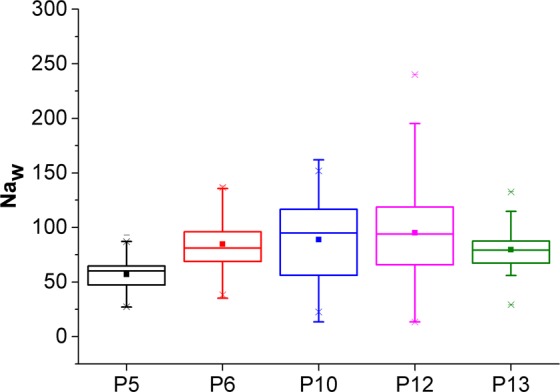
${[{\rm{Na}}]}_{{\rm{w}}}$ in the groundwater of the pristine catchment of Mule Hole. Bars correspond to the temporal variability of water composition in each piezometer (2006–2017).

In the Mule Hole forest, the concentration factor due to evapotranspiration (CF) calculated from Eq. (1) was 12.3 ± 5.64. This is consistent with the water balances calculated for this catchment^19,62^. The variability is probably due to variations in regolith depth and vegetation cover. Estimated ${[{\rm{Na}}]}_{w}$ was 80.72 ± 31.5 µmolL^−1^ and the spatial variability between piezometers was small compared to the temporal variability for each piezometer (Fig. 5). This suggests that plagioclase weathering depends more on temporal variations in residence time of water in the vadose zone than on the heterogeneity in bedrock composition^47^.

In the tubewells of the Berambadi catchment, using the average value of ${[{\rm{Na}}]}_{w}$ estimated in Mule Hole from Eq. 6, estimated CF was 34.7 ± 21.09, i.e. on average about 3 times more than in the natural system and with larger variability. We can explain this variability by the diversity in farming systems, with rainfed systems likely to have a low CF while irrigated systems likely to have high CF, because of longer duration crops and groundwater recycling.

**Figure 6:**
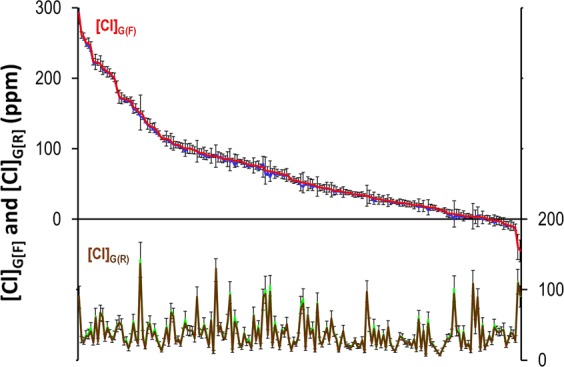
Contribution of rain [Cl]_G(R)_ (brown line) and fertilizers [Cl]_G(F)_ (red line) to the Cl concentration in groundwater in each tube wells of the Berambadi Catchment (ranked by decreasing [Cl]_G(F)_) calculated using the average [Na]_w_ in the forested catchment. The blue and green lines show respectively the average of [Cl]_G(R)_ and [Cl]_G(F)_ calculated using 20 random values spanning the range of the natural variability ${[{\rm{Na}}]}_{{\rm{w}}}$ and error bars show the standard deviation.

We deconvoluted Cl sources for the 188 tubewells of Berambadi using Eq. 12 for [Cl]_G(R)_ and Eq. 13 for [Cl]_G(F)_ and found that the average contribution of rain to the Cl concentration in groundwater [Cl]_G(R)_ was 38.1 ± 23.2 ppm, while the average contribution of fertilizers [Cl]_G(F)_ was 71.6 ± 67.78 ppm, i.e about 60% of the total concentration. Tubewells span a broad range of [Cl]_G(F)_ values from zero to 300 ppm (Fig. 6). While only 3% of the tubewells displayed slight negative values, suggesting that ${[{\rm{Na}}]}_{w}$ used in the analysis was not overestimated, 27% were above 100 ppm and could be noted as “hot spots. We performed a sensitivity analysis by using the deconvolution method for each tube well using 20 random ${[{\rm{Na}}]}_{w}$ values between 50 and 110 µmolL^−1^, that corresponds to the standard deviation of the natural variability observed in Mule Hole. The resulting variation of [Cl]_G(F)_ for each tubewell (error bars in Fig. 6) was small compared to the total range of variation, suggesting that the uncertainty in ${[{\rm{Na}}]}_{w}$ estimation is not likely to affect much the ranking of tubewells. It is noteworthy that [Cl]_G(R)_ and [Cl]_G(F)_ were not correlated, suggesting that variations in evapotranspiration are not the main/sole factor controlling the occurrence of hot spots of [Cl]_G(F)_.

### Spatial variability of groundwater quality

**Figure 7:**
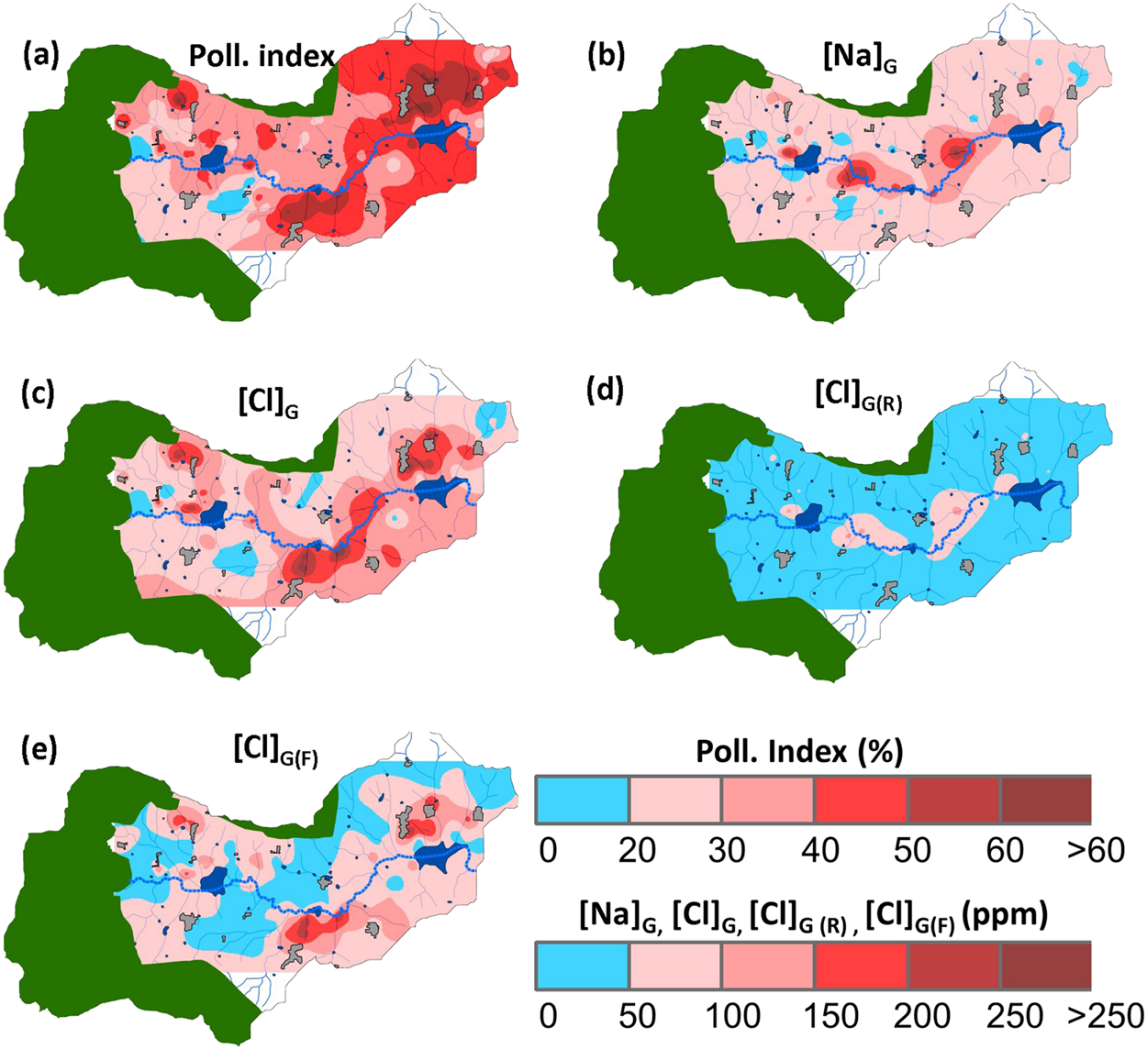
Spatial distribution of groundwater quality in the Berambadi catchment: (**a**) pollution index, concentration (ppm) in groundwater of (**b**) Sodium, (**c**) Chloride, (**d**) Contribution of rain [Cl]_G(R)_ and (**e**) fertilizers [Cl]_G(F)._ The green, dark blue and grey colours show forest, ponds and settlements respectively, the dotted blue line shows the main stream (maps were generated using ArcGIS version 10.3.0, https://desktop.arcgis.com/en/).

If groundwater quality displays large small-scale spatial heterogeneity, yet patterns can be observed at the landscape scale (Fig. 7). The pollution index (Fig. 7a) is increasing from the West, where values are close to those found in the pristine watershed, to the East where it is generally above 50%, with a hot spot close to the outlet. This latter area was identified as a vulnerable zone by^37^ due to the combination of groundwater depletion, low hydraulic gradient and intensive agricultural practices. It was also in the same area that significant impact of salinity on crop yield has been identified^38^. Na concentrations (Fig. 7b) were rather homogeneous, with hotspots located only along the main valley bottom. The spatial pattern of groundwater total Chloride concentration (Fig. 7c) is similar to the one of the Pollution Index, confirming that Chloride is a marker of groundwater quality degradation. The contribution of rain [Cl]_G(R)_ to Cl groundwater concentration is overall low (Fig. 7d). Largest values occur only in the valley bottoms, suggesting that these zones are prone to higher evapotranspiration rates. To the contrary, the contribution of fertilizers [Cl]_G(F)_ is higher on the hillslopes, with several hot spots (Fig. 7e).

## Discussion

Our analysis of the high-density tubewell sampling in the Berambadi agricultural catchment shows that the groundwater chemical composition displays high values of Chloride concentrations with a high small-scale spatial variability. In the following sections we first discuss the deconvolution method validity and robustness, then, the processes controlling the variability of concentration factor and Chloride sources in Berambadi. Finally, we highlight some implications of our findings for mitigating the observed incipient groundwater salinization.

### Validity assessment of deconvolution method

The deconvolution method we introduced for deconvoluting Chloride concentrations in groundwater using Na as a reference is based on three main assumptions: (1) dissolved Na and Cl are conservative, (2) they are subjected to the same concentration factor due to ET and 3) the release of Na by plagioclase weathering is similar for the pristine and the agricultural catchments.

#### Na and Cl are conservative

It is widely accepted that Chloride behaviour in catchments is conservative^52^ except in the presence of evaporite mineral formations. For Na, different processes can lead to non-conservative behaviour, and must be checked for specific sites. In our study site, previous studies demonstrated that Na is not recycled in any secondary mineral during weathering processes^23^ and only marginally adsorbed onto clays contrary to the other major cations Ca, Mg, K^73^. It was also recently demonstrated in the case of Mule Hole that Na was not significantly cycled through the vegetation^47,51^. This supports the absence of reactive behaviour of Na once in solution. In Berambadi, the same conservative behaviour is expected as the pedoclimatic and geological conditions are similar.

#### Concentration factor due to evapotranspiration is same for all Cl and Na sources

It is reasonable to assume that the concentration factor will be same for both sources of Cl, as both rain and fertilizers inputs are applied at the soil surface, and preferential flow is not likely to be significant in the study site context. For Na, while rain input is applied at the surface, inputs from weathering can occur at different depths. However, in the forested pristine catchment of Mule Hole it was recently demonstrated that plagioclase weathering occurs mostly in the soil and secondarily in the shallow underlying saprolite^51^ because the aggressiveness of solutions regarding primary silicate minerals decreases as solutions percolate in depth^23^. Evapotranspiration is also mostly happening in the soil layer and the shallow saprolite, as only few deep-rooted tree species access the deep vadose zone^63^, we can reasonably assume that in the forested pristine catchment, Na produced by weathering is subjected to similar CF as the rain inputs.

In contrast, in the agricultural context, characterized by shallow rooted crops, the sodium released by weathering below the root zone is not directly affected by evapotranspiration. However, larger evapotranspiration rate combined with recycling of groundwater for irrigation implies that the solutions infiltrating below the root zone are closer to saturation with respect to silicate minerals than in the forested watershed, and then less aggressive towards silicate minerals. As a consequence, the fraction of sodium released by weathering below the root zone is likely to be small. Neglecting it in Eq. 9 would lead to a slight underestimation of CF and subsequently a slight overestimation of [Cl]_G(F)_. In the present study, this possible bias may not have a large impact considering the range of [Cl]_G(F)_ found (Fig. 6), with a significant number of tubewell displaying [Cl]_G(F)_ values close to zero. In fact, in such groundwater irrigated systems, this bias is likely to be compensated by the additional evapotranspiration occurring during the multiple recycling of groundwater.

#### Similar release of Na by plagioclase weathering in pristine and agricultural catchments

The similar range of Eu* anomaly found in groundwater of both catchments suggests that plagioclase weathering intensity are similar in the pristine and the agricultural catchment. This might seem surprising as differences in weathering intensity are expected when groundwater residence time or to water aggressiveness are different. The residence time of water in the weathered zone is long in the forested catchment, because the significant water uptake by tree roots in the saprolite buffers groundwater recharge processes^62,63^. It ranges between 2 to more than 20 years depending on the saprolite thickness^47^. In Berambadi, plant uptake is limited to the root zone of crops and therefore the residence time of water in the vadose zone is likely to be shorter, not more than a few years. However, recycling of groundwater for irrigation increases the residence time of water and probably compensate at least partly the difference between the two catchments.

Weathering rates have been found to be higher in agricultural catchments than in pristine ones in temperate humid climate, mostly due to the acidification of soil pore water induced by large inputs of nitrogen fertilizers^53^. In Berambadi, groundwater pH is neutral and rather homogeneous across the catchment -even in nitrate-rich groundwater, because pore water solutions are buffered by the carbonate minerals in the regolith^73,74^ and in the bedrock^23^. Therefore, we can assume that fertilizer application does not significantly impact the intensity of plagioclase weathering in the catchment. Saturation indices calculated for each tube well using PHREEQC software (Fig. S1) show that while groundwater in Mule Hole is undersaturated with respect to albite (−2.5 ± 1), in Berambadi it is near equilibrium (−0.8 ± 0.6). According to^75^, plagioclase dissolution rate remains constant when saturation index varies between −4 to −0.5, supporting the assumption that Na_w_ estimated in the forest can be used for most of tube wells in the agricultural catchment. The spatial distribution of saturation index (Fig. S1) reveals that only few spots display saturation index beyond −0.5, mostly in the valley bottoms. Interestingly, these hot spots do not match the Na concentration ones (Fig. 7b). In locations with SI close to 0, the current plagioclase weathering rates could be limited. If this is the case, using the average Na_w_ estimated in the pristine catchment in these few locations would lead to an underestimation of CF and therefore an overestimation of the Cl added by fertilizers. However, the groundwater with high SI values did not display Eu-anomalies significantly different from those with low values, suggesting that average weathering rates were not different in these locations.

Importantly, we have shown that the deconvolution method was little sensitive to the estimated Na release from Na-plagioclase weathering (Fig. 3) mostly because, in the study case, Cl concentrations spanned over about 2 orders of magnitude, while Na_w_ variability is much lesser (Fig. 5).

### Variability of the concentration factor and sources of Chloride in the agricultural catchment

**Figure 8:**
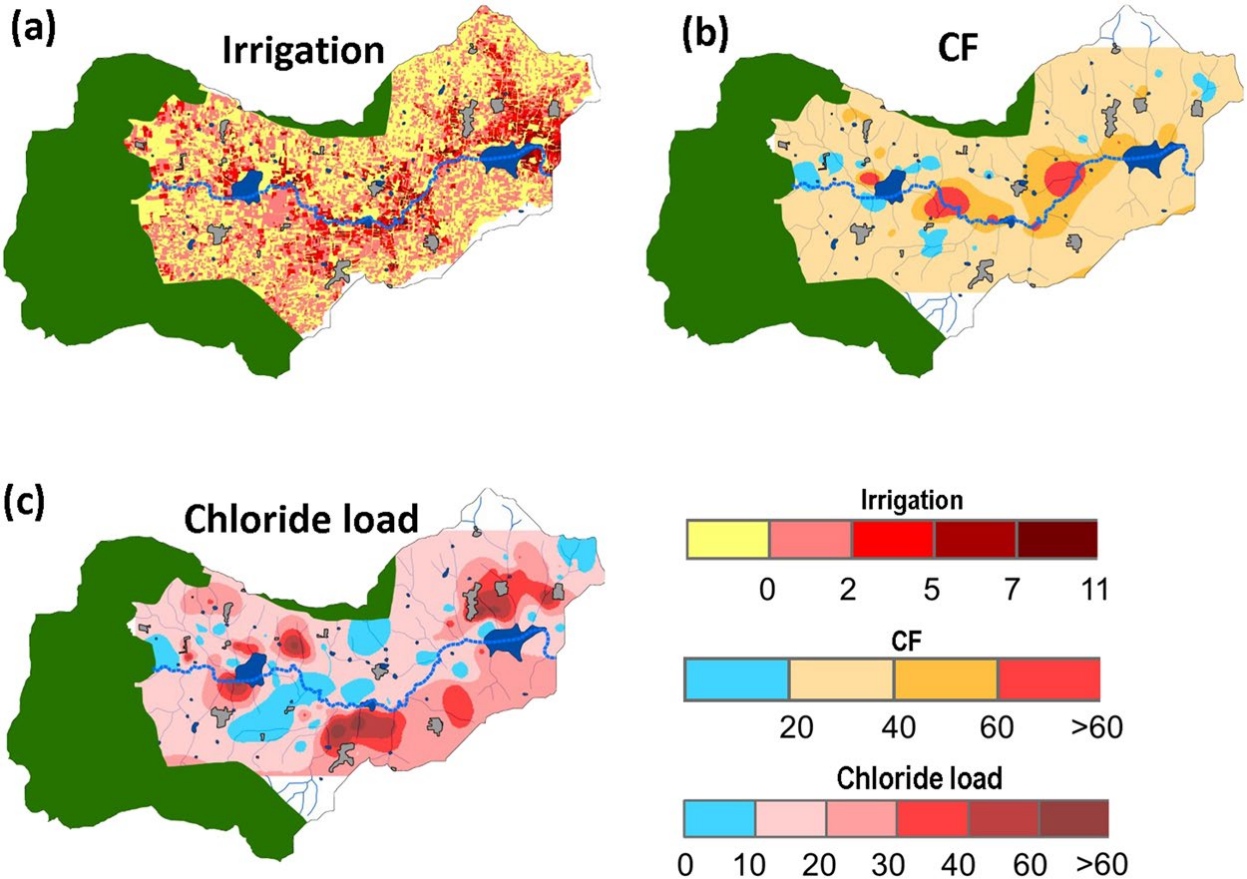
Spatial variations of (**a**) number of irrigation occurrences from 1990 to 2013 using satellite imagery (See methods) (**b**) Concentration factor due to evapotranspiration (CF, dimensionless) in the Berambadi catchment and (**c**) Chloride load (kg Cl. ha^−1^. y^−1^) originating from fertilizers. The green, dark blue and grey colours show forest, ponds and settlements respectively, the dotted blue line shows the main stream (maps were generated using ArcGIS version 10.3.0, https://desktop.arcgis.com/en/).

The concentration factor in the Berambadi groundwater was on average about 4 times greater than in the nearby forest of Mule Hole, suggesting that the proportion of rainfall contributing to the groundwater recharge is much lesser in the agricultural context. This can be explained on one hand by the lower annual rainfall in Berambadi (~800 mm) compared to Mule Hole (~1100 mm), and on the other hand by the widespread occurrence of groundwater irrigation across the catchment (Fig. 8a). Indeed, in addition to the enhanced evapotranspiration due to long or multiple crop cycles, groundwater irrigation is likely to induce higher concentration factor due to groundwater recycling^34^. In few hot spots, all located in the valley bottom along the streambed, the concentration factor was more than 60 (Fig. 8b). These zones are dominated by deep vertisols which low permeability favours intense evapotranspiration. In these soils, deep soil cracks favour high evaporation at depth, which can lead to a “desiccation-crack-induced salinization”^76^. However, in Berambadi, the largest concentrations of Cl originating from rain inputs ([Cl]_R_) were only about 150 ppm (Fig. 7d), which is not enough to impact soil and crop health. In the rest of the catchment, [Cl]_R_ remains very low, suggesting that rain Cl input alone, even in this context of semi-arid agriculture with large CF, would not induce salinization. To the contrary, large Cl concentrations originating from fertilizer inputs ([Cl]_G(F)_) were widespread in the hillslopes, with values up to 290 ppm (Fig. 7e).

The average relative contribution of [Cl]_G(F)_ to the groundwater concentration of the tubewells was 60%. Considering that average rainfall in Berambadi is 800 mm with Cl concentration of 1 ppm, the annual load from rainfall is about 8 kg Cl. ha^−1^. y^−1^. If this load represents 40% of the total Cl input, we can estimate that the average load from fertilizers was 20.1 ± 19.9 kgCl.ha^−1^. y^−1^, with a range between 0 and 90 kg Cl. ha^−1^yr^−1^. The spatial distribution of fertilizer load (Fig. 8c) shows that the practice of applying KCl fertilizers is widespread, with hot spots occurring in several regions, mostly in the hillslopes. Given the high small-scale spatial heterogeneity of practices and lateral groundwater flow, it is challenging to obtain independent assessment of fertilizer input contributing to individual wells. However, we found that our estimated range of inputs is consistent with available regional statistics: For example, at the scale of Karnataka state^77^ the estimated average K input in cultivated land in 2014–2015 is 32 kg. ha^−1^. y^−1^ (i.e. 29 kg Cl. ha^−1^. y^−1^ as virtually all the K is added in the form of KCl) which is slightly higher than the average input we found in Berambadi. Large variations are also found across cropping systems. For example, the 2001–2002 regional fertilizer statistics in Karnataka^78^ shows that K inputs is two times higher for irrigated crops compared to rainfed crops. This suggests that the incipient salinization we found in the Berambadi catchment, and its high spatial variability, is likely to be representative of the region.

### Implications for agricultural management for mitigation of incipient salinization

The Cl concentrations measured in the Berambadi groundwater reach values that are already affecting crop yield. In a recent study in the Berambadi catchment^38^, observed an inverse relationship between turmeric crop yield and soil pore-water conductivity, itself related to the conductivity of the irrigation groundwater. The results of the deconvolution method can provide useful information on the processes governing this incipient salinization, and help designing adequate mitigation policies.

For example, we showed natural features (namely soil properties) leading to high Concentration Factors induced larger values of Cl concentration in the valley bottoms. Even though the resulting concentrations has not reached threatening values, these zones appear to be very vulnerable to any change in agricultural practice that would either increase CF (such as increasing crop cycles or recycling groundwater for irrigation) or Cl inputs from fertilizers. Therefore, in such context, groundwater irrigation should be limited, or conjunctive use of ground and surface water should be promoted. In any case, addition of any form of fertilizer containing Cl should be strongly discouraged.

To the contrary, in the hillslopes, fertilizer addition is the main source of groundwater salinization, which can reach large values in the context of semi-arid agriculture with relatively high concentration factors due to evapotranspiration and groundwater recycling. In such context, mitigation action focused only on promoting practices that would decrease CF, i.e. either promoting rainfed agriculture or decrease water use efficiency of irrigated systems are unlikely to be successful. Finding alternative to KCl as potassium fertilizer would probably be more efficient. Alternatives, such as potassium silicate minerals (K-feldspar, micas) have been proposed which could both reduce salinization and fertilizer cost for the farmers^27^. However, research is needed to ensure both their agronomic and economic viability in semi-arid conditions.

## Conclusion

In this paper, we proposed a methodology to estimate the relative contribution of rainfall vs fertilizer addition to the total Cl concentration in groundwater and quantify the Concentration Factor due to evapotranspiration, using a neighbouring pristine catchment as a reference. We tested the validity of the deconvolution method in our study case by assessing the underlying assumptions, using previous studies in the same site and a geochemical proxy (Eu anomaly). We also assessed the robustness of the method by evaluation the error due to the uncertainty in weathering rate estimation. Applying the deconvolution method to other contexts would require the same careful testing the validity of its underlying assumptions.

We found that Potassium fertilization in the form of KCl is the main source of incipient salinization in the Berambadi catchment. However, sources and processes driving salinization in groundwater were found to vary spatially, with natural features favouring large concentration factor in valley bottoms while fertilization practices being dominant in the hillslopes. These results have strong relevance for designing efficient mitigation policies that should take into account these dominant drivers. The contribution of Cl input by fertilizer to groundwater salinization might vary according to contexts, depending on evapotranspiration intensity and agricultural practices affecting the concentration factor. As groundwater irrigation is fast developing in semi-arid to arid conditions, we expect them to be much more vulnerable to this salinization process than our study site under sub-humid climate. Therefore, we recommend that research on viable alternative forms of K should be urgently engaged to replace the widespread use of KCl for Potassium fertilization in groundwater irrigated semi-arid to arid zones.

## Supplementary information

Supplementary information.

## Data Availability

The datasets generated during and/or analysed during the current study are available from the authors on reasonable request.
