# Supplementary material for: Potash fertilizer promotes incipient salinization in groundwater irrigated semi-arid agriculture
Source: Sci Rep. 2020 Feb 28;10:3691. doi: 10.1038/s41598-020-60365-z (PMC7048856; doi:10.1038/s41598-020-60365-z)
Supplement: Supplementary file 1 — Supplementary information. [file 41598_2020_60365_MOESM1_ESM.docx]

**Potash fertilizer promotes incipient salinization in groundwater irrigated semi-arid agriculture**

Sriramulu Buvaneshwari, Jean Riotte, Muddu Sekhar, Amit Kumar Sharma, Rachel Helliwell, Mohan Kumar, M. S., J.J. Braun, Laurent Ruiz

*.
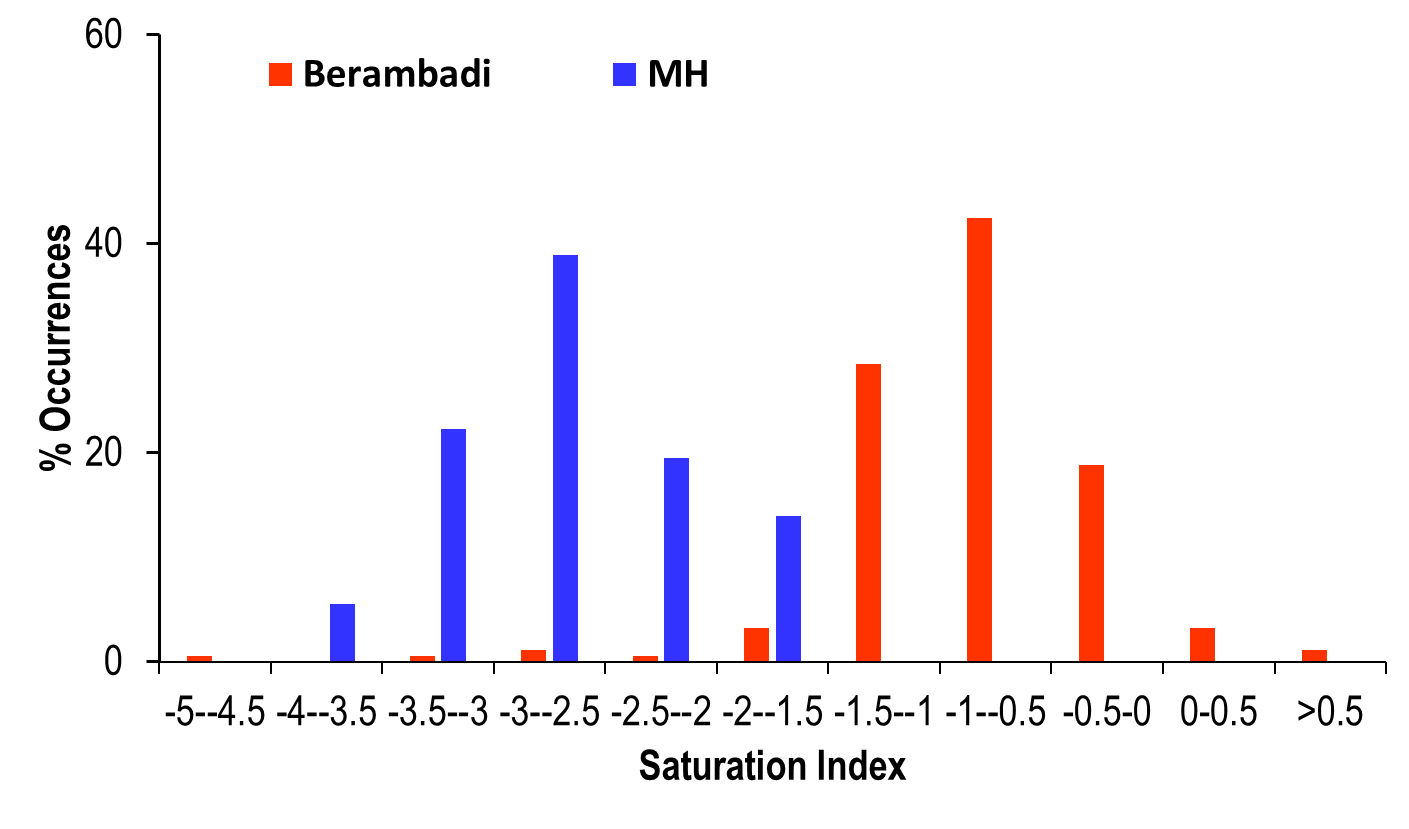
*

**Figure S1: Distribution of Saturation Index in Berambadi and Mule Hole**
